# 3,5-Dicarboxypyridinium fluoride

**DOI:** 10.1107/S1600536811021593

**Published:** 2011-06-18

**Authors:** Seik Weng Ng, Yi-Ping Tong

**Affiliations:** a Department of Chemistry, University of Malaya, 50603 Kuala Lumpur, Malaysia; b Department of Chemical Engineering, Huizhou University, Huizhou 516007, People’s Republic of China

## Abstract

The cation of the title salt, C_7_H_6_NO_4_^+.^F^−^, lies on a twofold rotation axis that passes through the N and 4-C atoms of the pyridine ring; the carb­oxy­lic acid substituent features unambiguous carbon–oxygen single and double bonds. The fluoride ion is a hydrogen-bond acceptor to two hy­droxy and one amino groups, these O—H⋯F and N—H⋯F hydrogen bonds leading to the formation of a layer structure parallel to the *ab* plane. The F atom lies on a position of *2* site symmetry.

## Related literature

For the crystal structure of pyridine-3,5-dicarb­oxy­lic acid, see: Cowan *et al.* (2005 ▶); Takusagawa *et al.* (1973 ▶).
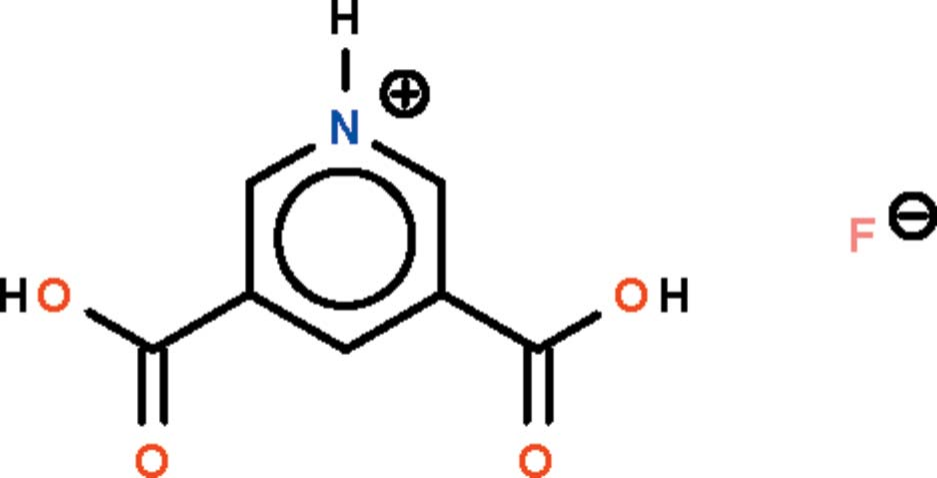


## Experimental

### 

#### Crystal data

C_7_H_6_NO_4_^+^·F^−^
*M* *_r_* = 187.13
Monoclinic, 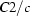

*a* = 11.3959 (14) Å
*b* = 11.4503 (14) Å
*c* = 6.1601 (7) Å
β = 104.197 (2)°
*V* = 779.26 (16) Å^3^
*Z* = 4
Mo *K*α radiation
μ = 0.15 mm^−1^
*T* = 293 K
0.40 × 0.35 × 0.25 mm

#### Data collection

Bruker SMART APEX diffractometer
Absorption correction: multi-scan (*SADABS*; Sheldrick, 1996 ▶) *T*_min_ = 0.686, *T*_max_ = 0.746
2354 measured reflections
883 independent reflections
750 reflections with *I* > 2σ(*I*)
*R*_int_ = 0.012

#### Refinement

*R*[*F* ^2^ > 2σ(*F* ^2^)] = 0.034
*wR*(*F* ^2^) = 0.105
*S* = 1.11
883 reflections
67 parameters
2 restraints
H atoms treated by a mixture of independent and constrained refinement
Δρ_max_ = 0.29 e Å^−3^
Δρ_min_ = −0.16 e Å^−3^

### 

Data collection: *APEX2* (Bruker, 2005 ▶); cell refinement: *SAINT* (Bruker, 2005 ▶); data reduction: *SAINT*; program(s) used to solve structure: *SHELXS97* (Sheldrick, 2008 ▶); program(s) used to refine structure: *SHELXL97* (Sheldrick, 2008 ▶); molecular graphics: *X-SEED* (Barbour, 2001 ▶); software used to prepare material for publication: *publCIF* (Westrip, 2010 ▶).

## Supplementary Material

Crystal structure: contains datablock(s) global, I. DOI: 10.1107/S1600536811021593/si2360sup1.cif

Structure factors: contains datablock(s) I. DOI: 10.1107/S1600536811021593/si2360Isup2.hkl

Supplementary material file. DOI: 10.1107/S1600536811021593/si2360Isup3.cml

Additional supplementary materials:  crystallographic information; 3D view; checkCIF report

## Figures and Tables

**Table 1:** Hydrogen-bond geometry (Å, °)

*D*—H⋯*A*	*D*—H	H⋯*A*	*D*⋯*A*	*D*—H⋯*A*
O1—H1⋯F1	0.86 (1)	1.60 (1)	2.458 (1)	176 (2)
N1—H2⋯F1^i^	0.88 (1)	1.68 (1)	2.563 (2)	180

Symmetry code: (i) 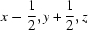.

**Fig. 1.**
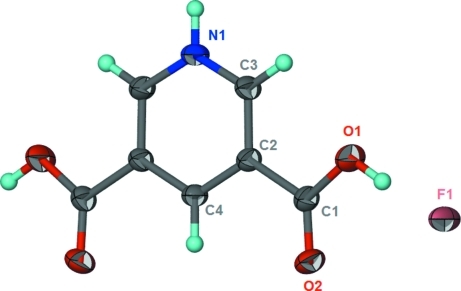
Thermal ellipsoid plot (Barbour, 2001) of C7H6NO4+ F- at the 50% probability level; hydrogen atoms are drawn as spheres of arbitrary radius. The unlabeled atoms are related to the labeled ones by –x, y, 3/2 - z.

**Fig. 2.**
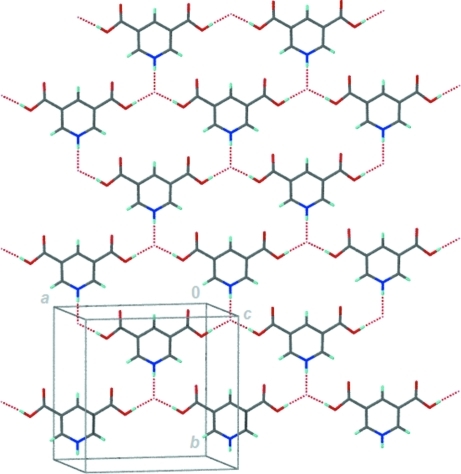
Layer structure.

## supplementary crystallographic information

### Comment

The organic salt was the crystalline product obtained in a hydrothermal reaction involving molybdic acid, hydrogen fluoride and pyridine-3,5-dicarboxylic acid; the reaction merely involved the protonation of the carboxylic acid by hydrogen fluoride. The parent carboxylic acid itself displays short O–H···O hydrogen bonds (Cowan *et al.*, 2005; Takusagawa *et al.*, 1973). The hydrogen fluoride salt, C_7_H_6_NO_4_^+^ F^-^ (Scheme I, Fig. 1), lies on a twofold rotation axis that passes through the pyridine ring; the carboxylic acid substituent features unambiguous carbon-oxygen single- and double-bonds [1.306 (1), 1.207 (1) Å]. The fluoride ion is hydrogen bond acceptor to two hydroxy and one amino groups, these O–H···F and N–H···F hydrogen bonds leading to the formation of a layer structure parallel to the *a*–*b* plane (Fig. 2).

### Experimental

To a solution of molybdic acid, H_2_MoO_4_ (1 mmol) in water (10 ml) was added 3,5-pyridinedicarboxylic acid (5 mmol). The mixture was placed in a 23 ml, Teflon-lined, stainless steel Parr bomb. Several drops of hydrofluoric acid were added. The bomb was heated at 373 for 3 days. It was then cooled to room temperature at 5 K per hour. Yellow block-shaped crystals were obtained in about 50% yield.

### Refinement

Carbon-bound H-atoms were placed in calculated positions (C—H 0.93 Å) and were included in the refinement in the riding model approximation, with *U*(H) set to 1.2*U*(C).

The amino and hydroxy H-atoms were located in a difference Fourier map, and were refined with a distance restraint of N–H 0.88±0.01 and O–H 0.84±0.01 Å; their temperature factors were freely refined.

### Crystal data

**Table:**

C_7_H_6_NO_4_^+^·F^−^	*F*(000) = 384
*M**_r_* = 187.13	*D*_x_ = 1.595 Mg m^−^^3^
Monoclinic, *C*2/*c*	Mo *K*α radiation, λ = 0.71073 Å
Hall symbol: -C 2yc	Cell parameters from 1129 reflections
*a* = 11.3959 (14) Å	θ = 2.6–28.4°
*b* = 11.4503 (14) Å	µ = 0.15 mm^−^^1^
*c* = 6.1601 (7) Å	*T* = 293 K
β = 104.197 (2)°	Block, yellow
*V* = 779.26 (16) Å^3^	0.40 × 0.35 × 0.25 mm
*Z* = 4	

### Data collection

**Table:**

Bruker SMART APEX diffractometer	883 independent reflections
Radiation source: fine-focus sealed tube	750 reflections with *I* > 2σ(*I*)
graphite	*R*_int_ = 0.012
ω scans	θ_max_ = 27.5°, θ_min_ = 2.6°
Absorption correction: multi-scan (*SADABS*; Sheldrick, 1996)	*h* = −10→14
*T*_min_ = 0.686, *T*_max_ = 0.746	*k* = −13→14
2354 measured reflections	*l* = −8→5

### Refinement

**Table:**

Refinement on *F*^2^	Primary atom site location: structure-invariant direct methods
Least-squares matrix: full	Secondary atom site location: difference Fourier map
*R*[*F*^2^ > 2σ(*F*^2^)] = 0.034	Hydrogen site location: inferred from neighbouring sites
*wR*(*F*^2^) = 0.105	H atoms treated by a mixture of independent and constrained refinement
*S* = 1.11	*w* = 1/[σ^2^(*F*_o_^2^) + (0.0615*P*)^2^ + 0.1524*P*] where *P* = (*F*_o_^2^ + 2*F*_c_^2^)/3
883 reflections	(Δ/σ)_max_ = 0.001
67 parameters	Δρ_max_ = 0.29 e Å^−^^3^
2 restraints	Δρ_min_ = −0.16 e Å^−^^3^

### Fractional atomic coordinates and isotropic or equivalent isotropic displacement parameters (Å^2^)

**Table:**

	*x*	*y*	*z*	*U*_iso_*/*U*_eq_	
F1	0.5000	0.54662 (9)	0.7500	0.0536 (4)	
O1	0.30965 (8)	0.64863 (8)	0.74566 (19)	0.0454 (3)	
H1	0.3745 (12)	0.6097 (17)	0.747 (3)	0.069 (6)*	
O2	0.21589 (9)	0.47552 (8)	0.71733 (17)	0.0428 (3)	
H2	0.0000	0.8997 (9)	0.7500	0.050 (6)*	
N1	0.0000	0.82283 (12)	0.7500	0.0346 (4)	
C1	0.21620 (10)	0.58059 (11)	0.7326 (2)	0.0321 (3)	
C2	0.10381 (10)	0.64621 (10)	0.73953 (19)	0.0294 (3)	
C3	0.10135 (10)	0.76662 (11)	0.7394 (2)	0.0328 (3)	
H3	0.1701	0.8087	0.7320	0.039*	
C4	0.0000	0.58621 (14)	0.7500	0.0294 (4)	
H4	0.0000	0.5050	0.7500	0.035*	

### Atomic displacement parameters (Å^2^)

**Table:**

	*U*^11^	*U*^22^	*U*^33^	*U*^12^	*U*^13^	*U*^23^
F1	0.0272 (6)	0.0273 (6)	0.1130 (11)	0.000	0.0301 (6)	0.000
O1	0.0245 (5)	0.0335 (5)	0.0811 (7)	0.0017 (4)	0.0183 (5)	−0.0042 (5)
O2	0.0370 (6)	0.0281 (5)	0.0663 (7)	0.0059 (4)	0.0184 (5)	−0.0033 (4)
N1	0.0268 (7)	0.0209 (7)	0.0570 (9)	0.000	0.0121 (6)	0.000
C1	0.0265 (6)	0.0299 (6)	0.0409 (7)	0.0029 (5)	0.0102 (5)	−0.0008 (5)
C2	0.0249 (6)	0.0254 (6)	0.0385 (6)	0.0012 (4)	0.0087 (5)	−0.0012 (4)
C3	0.0239 (6)	0.0260 (6)	0.0495 (7)	−0.0025 (4)	0.0108 (5)	−0.0004 (5)
C4	0.0272 (8)	0.0218 (7)	0.0394 (9)	0.000	0.0085 (6)	0.000

### Geometric parameters (Å, °)

**Table:**

O1—C1	1.306 (1)	C1—C2	1.495 (2)
O1—H1	0.86 (1)	C2—C3	1.379 (2)
O2—C1	1.207 (2)	C2—C4	1.383 (1)
N1—C3^i^	1.338 (1)	C3—H3	0.9300
N1—C3	1.338 (1)	C4—C2^i^	1.383 (1)
N1—H2	0.88 (1)	C4—H4	0.9300
			
C1—O1—H1	112.1 (14)	C3—C2—C1	121.33 (11)
C3^i^—N1—C3	122.48 (15)	C4—C2—C1	120.03 (11)
C3^i^—N1—H2	118.76 (7)	N1—C3—C2	119.92 (11)
C3—N1—H2	118.76 (7)	N1—C3—H3	120.0
O2—C1—O1	125.90 (11)	C2—C3—H3	120.0
O2—C1—C2	121.15 (11)	C2—C4—C2^i^	120.44 (15)
O1—C1—C2	112.95 (11)	C2—C4—H4	119.8
C3—C2—C4	118.62 (11)	C2^i^—C4—H4	119.8
			
O2—C1—C2—C3	−174.90 (12)	C4—C2—C3—N1	0.20 (16)
O1—C1—C2—C3	5.39 (16)	C1—C2—C3—N1	−178.73 (9)
O2—C1—C2—C4	6.18 (17)	C3—C2—C4—C2^i^	−0.10 (8)
O1—C1—C2—C4	−173.53 (9)	C1—C2—C4—C2^i^	178.85 (11)
C3^i^—N1—C3—C2	−0.10 (8)		

Symmetry codes: (i) −*x*, *y*, −*z*+3/2.

### Hydrogen-bond geometry (Å, °)

**Table:**

*D*—H···*A*	*D*—H	H···*A*	*D*···*A*	*D*—H···*A*
O1—H1···F1	0.86 (1)	1.60 (1)	2.458 (1)	176 (2)
N1—H2···F1^ii^	0.88 (1)	1.68 (1)	2.563 (2)	180

Symmetry codes: (ii) *x*−1/2, *y*+1/2, *z*.
